# Impact of an Obstetrical Emergency Simulation Learning Module on Midwifery Students' Competency: A Non-randomized Control Study

**DOI:** 10.7759/cureus.81532

**Published:** 2025-03-31

**Authors:** Saraswathy Kannappan, Susila Chandrasekaran, Latha Venkatesan, Merlin Ashok

**Affiliations:** 1 Nursing: Obstetrics and Gynecology, Apollo College of Nursing, Affiliated With The Tamil Nadu Dr. MGR Medical University, Chennai, IND; 2 Nursing, Billroth College of Nursing, Chennai, IND; 3 Obsterics and Gynaecology, All India Institute of Medical Sciences, New Delhi, New Delhi, IND; 4 Nursing, Apollo College of Nursing, Affiliated With The Tamil Nadu Dr. MGR Medical University, Chennai, IND

**Keywords:** competency, knowledge, learning experience, midwifery students, obstetrical emergencies, postpartum hemorrhage (pph), simulation, simulation based learning, simulation learning module, skills

## Abstract

Background

Obstetrical emergencies are multifaceted and complex, and their urgency demands a combination of clinical skills. As a result, it is generally observed that minimum teaching takes place during these emergencies. This study aimed to formulate a simulation learning module centered on chosen obstetrical emergencies and evaluate its efficacy in measuring the competency of undergraduate midwifery students.

Design and participants

The study utilized a non-randomized time series interventional research design among 188 midwifery students (control group 90, intervention group 98). A hybrid simulation was administered to the intervention group. The research employed several tools: a proforma for background variables, a structured questionnaire, and the Objective Structured Clinical Examination (OSCE) of selected obstetrical emergencies.

Results

In the present study, 98% of participants in the control group demonstrated inadequate competency in the pretest, post-test I, and post-test II. In contrast, all participants (100%) in the intervention group had adequate competency followed by moderately adequate competency in the post-test I and post-test II. The study reported that there was a significant difference between the assessments (pretest, post-test-I, post-test-II) in the knowledge and skills of the interventional group with the effect size (eta squared=0.70, 0.99), respectively. The results showed a statistically significant difference (P<0.001) in overall competency scores between the control and intervention groups at pretest, with a moderate effect size (Cohen's d=0.68). The intervention group demonstrated substantial improvements at post-test I (Cohen's d=23.61) and post-test II (Cohen's d=15.83).

Conclusion

Hybrid simulation is an appropriate method for equipping healthcare providers to gain and maintain competence in managing obstetric emergencies. Therefore, simulation must be incorporated into the curriculum to allow students to immerse themselves in their clinical learning, which enhances their communication, confidence, and satisfaction.

## Introduction

Obstetrical emergencies are a paramount concern in midwives’ daily practice. According to the United Nations Maternal Mortality Estimation Inter-agency Group (UN MMEIG) 2020 report, "Trends in maternal mortality 2000 to 2020," the global maternal mortality ratio (MMR) declined from 339 in 2000 to 223 in 2020, representing an average annual rate of reduction (ARR) of 2.1%. In contrast, India's MMR decreased from 384 to 103 during the same period, corresponding to an ARR of 6.36%, which is higher than the global average [1]. Additionally, the World Factbook (2020) ranks India 68th among 186 nations in terms of maternal mortality, with an MMR of 103 per 100,000 live births [2]. The management of obstetric emergencies requires organized teamwork in which the competency of the midwives plays the role of a keystone.

Nagarajan R, in a news article, quotes that among the sustainable development goals (SDGs), goal 3 has the target of 3.1 in which, by 2030, the global MMR ratio should be less than 70 per 100000 live births by achieving 97 in 2018-2020 [3]. Quality of care in emergency obstetrics involves institutional and staff preparedness in the provision of appropriate emergency services while responding to the needs and rights of the clients. Being the primary health care providers, the preparedness of nurses for obstetric emergencies attains paramount importance. The challenges perceived by them in managing those urgent situations demand immediate interventions to ensure safe pregnancy and childbirth for the obstetric population. Furthermore, conventional teaching methods (e.g., didactic courses, guides, and manuals) have proven to be of limited effectiveness in promoting the adoption of evidence-based practices by midwives.

Clinical teaching is aimed at producing a competent registered nurse capable of providing nursing care that is based on sound knowledge and practical skills. While most midwifery educators today are baby boomers, the majority of midwifery students are millennial and generation-Z individuals who communicate, learn, and interact very differently from their instructors. A variety of educational practices are useful when incorporating simulation into nursing education programs. By engaging learners directly in the simulation, active learning occurs.

Saraswathy et al. also noted that about 68% of participants preferred simulation training as an effective method of teaching and learning obstetrical emergencies [4]. Parker explains that the integration of complex simulation can be a powerful learning tool in articulating the sophisticated integration of knowledge, skills, and attitudes [5].

The Health Management Information System (HMIS) (2020-21) measures maternal deaths caused due to hemorrhage (14%), hypertension (12%), obstructed or prolonged labor (3%), high fever (3%), abortion (2%), and unknown causes (66%) [6]. Many maternal deaths are caused by the failure of health providers to recognize post and pre-natal complications, delays in initiating appropriate treatment, and the lack of emergency preparedness at health facilities, as identified by CommonHealth and Jan Swasthya Abhiyan, both coalitions of health activists in India [7].

Mehdipour states in the study that the simulation-based mastery model used by the intervention group was significantly more effective than the traditional training used by the control. These findings showed that mastery learning effectively improves clinical skills among undergraduate nursing students [8]. A focus group study on simulation-based training promoted self-confidence, improved clinical skills and judgment in clinical practice, and emphasized the importance of communication and team collaboration. This study revealed students’ transfer of learning outcomes from simulation-based training to clinical practice [9].

The researcher observed a high percentage of incompetence in novice nurses during the clinical postings. They were hands-off in times of crisis, and when interviewed, they were afraid that they would harm the mother in an emergency. Any emergency care can’t be taught in real-time clinical experience; this can be done only through simulation. In this research endeavor, the investigator aimed to create and evaluate a simulation learning module that focuses on specific obstetrical emergencies relevant to midwifery students. The study's objective was to assess the impact of simulation-based training on nursing students’ competencies in managing obstetric emergencies.

## Materials and methods

Study design and participants

A non-randomized time series interventional research design was employed across three nursing colleges from June 2021 to May 2022. The sample size was determined based on pilot study findings, considering the competency of undergraduate midwifery students. The pilot study revealed standard deviations of 2.97 and 3.818 for the control and intervention groups, respectively, with a type I error (α) of 0.05, power (1-β) of 0.90, and an effect size of 0.45. The necessary sample size was estimated at 80 per group through the OpenEpi online calculator (https://www.openepi.com/). To mitigate the impact of an expected attrition rate of 10%, which corresponds to 8 participants, all available samples were included, yielding 92 students in the control group (46 from each college) and 100 in the intervention group. Post-attrition, the study comprised 98 students in the intervention group and 90 students (46 from one college and 44 from another) in the control group. Participants were chosen using a total enumerative sampling method.

Intervention

Based on the first objective, the researcher developed the Obstetrical Emergency Simulation Learning Module, aligned with the needs assessment study findings. The module included simulation scenarios for managing eclampsia, antepartum hemorrhage, cord prolapse, prolonged labor, and postpartum hemorrhage, which were refined with input from simulation experts. Five simulation scenarios, each lasting one hour, were conducted over 40 days for the intervention group. Each condition was practiced by two groups (six members per group) per day over eight days. Sessions were held at the Apollo Simulation Centre, utilizing moderate-fidelity mannequins (Mamma Birthie, Mamma Natelle (Laerdal Medical, Stavanger, Norway) and standardized patient role-playing. Each scenario was pre-briefed for 5-7 minutes, followed by an 8-10 minute performance, and concluded with a 20-40 minute debriefing using the CORE and Plus Delta models. Students reflected on their performance, essential practices were reinforced, and feedback was provided as needed to enhance learning outcomes.

Instruments and other variables

 The researcher developed tools to assess various background variables, including age, native state, medium of instruction in higher secondary education, prior knowledge of simulation-based learning, and third-year B.Sc. Nursing examination scores. Competency in managing obstetrical emergencies was evaluated using a structured knowledge questionnaire and an OSCE skills checklist (Appendix A). The structured questionnaire, designed to assess understanding of obstetrical emergencies, demonstrated high internal consistency (r=0.9). It comprised 30 multiple-choice questions, with each correct answer awarded one mark, and scores were categorized as adequate (75-100%), moderately adequate (50-74%), or inadequate (<50%). The OSCE checklist, developed to evaluate students’ practical skills, exhibited good reliability (r=0.9) through interclass coefficient testing.

Trained evaluators assessed student performance across 16 OSCE stations, covering eclampsia, antepartum hemorrhage, cord prolapse, prolonged labor, and postpartum hemorrhage (Appendix B). Additionally, a rating scale on satisfaction with simulation-based learning was developed, showing good reliability (r=0.8) through Cronbach's alpha (Appendix C). The self-administered scale consisted of 15 items evaluating the researcher’s characteristics, the simulation module, and the effectiveness of the method, with responses rated from highly satisfied (4) to highly dissatisfied (1). The total score (60) was interpreted into four levels of satisfaction. The analysis of the collected data was conducted using SPSS Version 23 (IBM Corp., Armonk, NY). Descriptive statistics, such as frequency, percentage, mean, standard deviation, Cohen’s d for effect size in independent t-tests, and eta squared for effect size in repeated measures analysis of variance (Rm-ANOVA) were applied. Inferential statistics included chi-square tests to evaluate the homogeneity of participants in both groups, analysis of variance (Rm-ANOVA with post-hoc comparisons) for assessing competency differences across assessments, and independent t-tests to compare competencies between the two groups.

Ethical considerations

The study was conducted after obtaining ethical clearance from the Institutional Ethics Committee (IEC) of Apollo College of Nursing, Chennai, with IEC No. ACON C/IEC/2018/018. Permission was obtained from the principals of selected nursing colleges. The purpose of the study was explained to the participants. Written consent was obtained from all participants before data collection. Confidentiality was maintained throughout the study. Debriefing was done after each session on simulation learning on obstetrical emergencies. Control group students were taught about the obstetrical emergency simulation module and simulation learning was conducted using the simulation scenario on obstetrical emergencies after data collection.

Data collection

Phase I

The researcher was trained in simulation at the National Simulation Reference Centre, SGT University, Gurgaon, organized by the Indian Nursing Council and Apollo Simulation Centre, Chennai, and completed the Sim Begin Course conducted by SAFER (Stavanger Acute Medicine Foundation for Education and Research) and Laederal Medical. The obstetrical emergency simulation learning module was developed by the researcher based on the needs assessment conducted with the help of experts in the fields of simulation and obstetrics and gynecology nursing [4]. It consists of the curriculum, algorithms, and simulation scenarios on the management of obstetrical emergencies. Simulation scenarios were developed based on templates from the WHO and the National League for Nurses (NLN).

Phase II

Students from two colleges were selected for the control group based on sample availability, while one college in Chennai was chosen for the intervention group. Prior to data collection, it was ensured that theory classes on obstetrical emergencies, delivered via lectures, Microsoft PowerPoint (Microsoft Corporation, Redmond, WA, US), and videos, were completed as prerequisites. Participants provided informed consent and completed a background proforma and structured questionnaire. OSCE skill stations were set up in maternity labs, and students were randomly assigned into three groups of 16 for pretest and post-test assessments. The intervention group received simulation learning facilitated by the researcher, who also developed the simulation scenarios per the intervention protocol. Post-test I and II were conducted using a structured questionnaire and OSCE stations. In the control group, post-test I was conducted 7 days after the pretest and post-test II after 30 days. In the intervention group, post-test I was conducted 7 days after simulation training, and post-test II after 30 days. Additionally, intervention group students assessed their satisfaction with simulation learning using a rating scale during post-test I.

## Results

Phase I

Simulation scenarios were focused on the learner-centered facilitative approach driven by the objectives, learners’ knowledge, and level of experience, and the expected outcomes intended from the Royal College of Obstetricians and Gynecologists (RCOG), American College of Obstetricians and Gynecologists (ACOG), Dakshata Guidelines by Ministry of Health and Family Welfare by Government of India [10], scope of midwifery practice by Indian Nursing Council [11], and WHO SBA guidelines [12]. Obstetrical emergencies like the eclampsia scenario were structured with four stages, antepartum hemorrhage with three stages, cord prolapse with four stages, prolonged labor with three stages, and postpartum hemorrhage (PPH) with five stages. Simulation scenarios were pilot-tested with the simulation experts before implementation for better validation.

Phase II

Table 1 depicts that most of them (100%, 86.7%) were aged between 21 to 22 years, 93.3% and 100% were female students, and 82.2% and 84.74% studied in the English language in higher secondary education in the control and intervention groups, respectively. More than half of the control group (53.3%) and 43.87% in the intervention group scored 60-74% marks in their third-year university exams. However, there was no statistically significant difference between the control and Intervention groups (P>0.05). Therefore, both the groups were homogenous and comparable except age and gender.

**Table 1:** Frequency and percentage distribution of background variables of the undergraduate midwifery students (n=188)

Background Variables	Control Group (n=90)		Intervention Group (n=98)		χ^2^ value/	P value
	f	%	f	%		
Age in Years						
18-20	0	0	13	13.3	NA	
21-22	90	100	85	86.7		
Gender						
Male	6	6.7	0	0	NA	
Female	84	93.3	98	100		
Medium of Instruction (Up to Hr. Sec.)						
Tamil	16	17.8	15	15.3	0.208	0.648
English	74	82.2	83	84.74		
Percentage of Marks in IIIrd Year						
75-100	42	46.7	55	56.12	1.680	0.195
60-74	48	53.3	43	43.87		
50-59	0	0	0	0		

Table 2 indicates that in the control group, the majority had moderately adequate knowledge in the pretest (58.7%), post-test I (60%), and post-test II (63.3%). In contrast, the intervention group showed an improvement, with 52% having moderately adequate knowledge in the pretest, 53.1% achieving adequate knowledge in post-test I, and 62.2% having moderately adequate knowledge in post-test II. Regarding skills, all students (100%) in both groups had inadequate skills in the pretest. However, after simulation training, the intervention group showed significant improvement, with 100% achieving adequate skills in post-test I and 74.5% maintaining moderately adequate skills in post-test II. Competency levels followed a similar trend, with 98% of the control group maintaining moderately adequate competency throughout, whereas the intervention group improved from 100% moderately adequate in the pretest to 100% adequate in post-test I and II. These results reinforce the effectiveness of simulation training in enhancing knowledge, skills, and competency in managing obstetrical emergencies.

**Table 2:** Frequency & percentage distribution of background variables of the undergraduate midwifery students (n=188)

Levels	Control Group (n=90)						Intervention Group (n=98)					
	Pretest		Post-test I		Post-test II		Pretest		Post-test I		Post-test II	
	f	%	f	%	f	%	f	%	f	%	f	%
Level of Knowledge												
Adequate (75-100%)	0	0	0	0	0	0	0	0	52	53.1	34	34.7
Moderately Adequate (50-74%)	53	58.9	54	60	57	63.3	51	52	44	44.9	61	62.2
Inadequate <50%	37	41.3	36	40	33	36.7	47	48	2	2	3	3.1
Level of Skills												
Adequate (75-100%)	0	0	0	0	0	0	0	0	98	100	25	25.5
Moderately Adequate (50-74%)	0	0	0	0	0	0	0	0	0	0	73	74.5
Inadequate <50%	90	100	90	100	90	100	98	100	0	0	0	0
Level of Competency (Overall Knowledge & Skill)												
Adequate (75-100%)	0	0	0	0	0	0	0	0	98	100	23	23.5
Moderately Adequate (50-74%)	0	0	0	0	0	0	0	0	0	0	75	76.5
Inadequate <50%	90	100	90	100	90	100	98	100	0	0	0	0

Table 3 represents that in Rm-ANOVA, there was a statistically significant difference in knowledge in the pre-test, post-test I, and post-test II of the selected obstetrical emergencies like eclampsia, antepartum hemorrhage (APH), cord prolapse, prolonged labor, PPH, and total mean scores of the knowledge in the interventional group with the effect size (eta squared=0.70), skills in the control group (eta squared=0.42), and skills in the intervention group (eta squared= 0.99) were statistically significant between assessments (P<0.001). Hence, per Hypothesis H1, “There will be a significant difference in competency (Knowledge and Skill) on managing selected obstetrical emergencies between assessments in control and intervention group of undergraduate midwifery students”; the difference in the knowledge of the control group was partially accepted at P<0.001.

**Table 3:** Comparison of competency scores in pretest, post-test I, and post-test II between the control and intervention groups of undergraduate midwifery students (n=188) APH: antepartum hemorrhage; PPH: postpartum hemorrhage

Components	Control (n=90)		Intervention (n=98)		MD	Independent ‘t’ Value (P value)	Cohen’s d
	Mean	SD	Mean	SD			
Pre-test							
Eclampsia	9.35	1.40	8.56	2.10	-0.79	3.02 (0.003)	0.4 (0.20-0.78)
APH	13.26	1.66	12.12	1.46	-1.14	5.01 (<0.001)	0.72 (0.43-1.02)
Cord Prolapse	8.42	1.45	8.89	1.39	0.47	2.28 (0.02)	0.32 (0.61-0.04)
Prolonged labor	12.12	1.41	12.75	1.79	0.63	2.26 (<0.001)	0.38 (-0.67-0.09)
PPH	20.77	1.61	18.90	2.05	0.27	6.95 (<0.001)	1.00 (0.70-1.31)
Total	63.94	3.73	61.24	4.11	-2.69	-4.69 (<0.001)	0.68 (0.39-0.98)
Post-test-I							
Eclampsia	9.54	1.09	22.98	1.31	13.44	75.69 (<0.001)	11.06 ( 9.93-12.25)
APH	13.47	1.50	31.41	1.76	17.94	74.77 (<0.001)	15.22 (13.68-16.84)
Cord Prolapse	8.87	1.30	21.98	1.47	13.11	64.31 (<0.001)	9.38 (8.41-10.40)
Prolonged labor	12.38	1.33	31.18	1.74	18.79	82.39 (<0.001)	12.02 (10.79-13.31)
PPH	20.85	1.60	57.44	2.16	36.59	130.76 (<0.001)	19.05 (17.14-21.07)
Total Score	65.14	3.49	165.03	4.78	99.88	162.37 (<0.001)	23.61 (21.25-26.10)
Post-test-II							
Eclampsia	9.86	1.09	19.48	1.40	9.62	51.99 (<0.001)	7.59 (6.79-8.44)
APH	13.78	1.63	26.31	2.12	12.52	45.00 (<0.001)	6.56 (5.85-7.30)
Cord Prolapse	9.10	1.48	19.11	1.99	10.01	38.70 (<0.001)	5.65 (5.02-6.30)
Prolonged Labor	12.55	1.35	26.36	1.80	13.81	58.95 (<0.001)	8.59 (7.69-9.53)
PPH	20.82	1.59	44.72	2.40	23.90	79.60 (<0.001)	11.59 (10.41-12.84)
Total Score	66.13	3.71	136.01	4.94	134.02	257.05 (<0.001)	15.83 (14.23-17.52)

**Figure 1:**
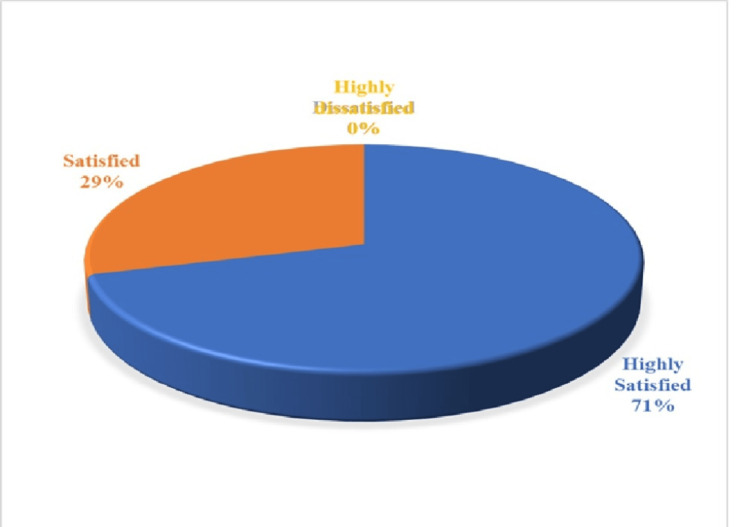
Percentage distribution of level of satisfaction on the Obstetrical Emergency Simulation Module

Table 3 also denotes that there was significant difference in pretest mean competency scores specific to the components like APH, PPH and overall competency scores at P<0.001 with the effect size (Cohen's d = 0.72, 1.00); however, in post-test I and post-test II, selected obstetrical emergencies like eclampsia, APH, cord prolapse, prolonged labor, PPH, and overall competency scores between the control and intervention groups were statistically significant at P<0.001 with a larger effect size (Cohen's d >0.8). Hence, Hypothesis H2, “There will be a significant difference in competency on managing selected obstetrical emergencies between the control and intervention groups of undergraduate midwifery students” was accepted. Figure 1 displays that most participants (71%) were highly satisfied, whereas only 29% were satisfied regarding the obstetrical emergency simulation learning in the Intervention group.

Table 4 displays a pairwise comparison of knowledge and skills on selected obstetrical emergencies between the assessments of pre-test vs. post-test I, post-test I vs. post-test II, and post-test II vs. pre-test, which were statistically significant (P<0.001) with 95% CI.

**Table 4:** Post-hoc analysis (pair-wise comparison) of knowledge and skill scores on obstetrical emergencies among the control and experimental group of undergraduate midwifery students (n=188)

Group	Assessments	Mean Difference	Standard Error	P value	95% CI	
					Lower Bound	Upper Bound
Knowledge						
Control Group	Pre-test vs. Post-test I	0.44	0.24	0.217	0.15	1.04
	Post-test I vs. Post-test II	0.022	0.23	1.000	0.55	0.59
	Pretest vs. Post-test II	0.422	0.05	<0.001	0.28	0.56
Interventional Group	Pre-test vs. Post-test I	8.918	0.44	<0.001	7.84	9.99
	Post-test I vs. Post-test II	9.408	0.50	<0.001	8.17	10.64
	Pretest vs. Post-test II	0.490	0.52	1.000	0.78	1.76
Skills						
Control Group	Pre-test vs. Post-test I	1.70	0.19	<0.001	1.22	2.17
	Post-test I vs. Post-test II	1.10	0.09	<0.001	0.86	1.33
	Pretest vs. Post-test II	0.60	0.13	<0.001	0.26	0.93
Interventional group	Pre-test vs. Post-test I	67.94	0.45	<0.001	66.83	69.06
	Post-test I vs. Post-test II	96.52	0.51	<0.001	95.26	97.77
	Pretest vs. Post-test II	28.57	0.52	<0.001	27.30	29.83

## Discussion

Phase I

The researcher developed the simulation scenarios. A similar study on the efficacy of simulation using the NLN/Jeffries Nursing Education Simulation Framework supported the components of the teaching-learning process and their relationships to guide the implementation and evaluation of these activities [13]. This study also highlights the need for standardized and structured simulation scenarios that comprehensively encompass all the facets of simulation design. Nurse educators need to develop evidence-based teaching skills that will help them to critically evaluate the evidence for content additions and deletions and decide what knowledge is essential for students to acquire.

Phase II

The present study found that the majority of participants (100%, 86.7%) were aged 21-22 years and had studied in the English medium (82.2%, 84.7%) for their higher secondary education. Over half of the control group (48; 53.3%) and 43 (43.87%) of the intervention group scored 60-74% in their third-year university exams. Similar studies have reported comparable demographics, with most students aged 21 years (63%), having English as their medium of instruction (78%), and with 59% achieving distinction [14]. While female students still dominate nursing education, the intake of male students is gradually rising in South India. Despite challenges, all students persevere to meet professional requirements in obstetrics, demonstrating a strong commitment to acquiring knowledge and skills.

The present study found that in the control group, most participants (98%) had moderately adequate competency in pre-test, post-test I, and post-test II. In contrast, all participants (100%) in the Intervention group had moderately adequate competency initially but demonstrated adequate competency in post-test I and post-test II. These findings align with a prospective study evaluating the impact of simulation-based training on obstetrical emergencies, where 87% of participants reported improved knowledge and skills in real emergencies. Obstetric nurses showed significantly higher improvements than obstetricians in diagnosing emergencies (p=0.002), technical skills (p=0.024), teamwork (p=0.005), and knowledge of management guidelines (p=0.006) [15]. Similarly, simulation-based training has been shown to enhance clinical skills, decision-making, communication, and team collaboration, reinforcing the transfer of learning from simulation to clinical practice [9].

The study reported that there was a significant difference between the assessments (pretest, post-test-I, post-test-II) in the knowledge and skills of the interventional group with the effect size of eta squared=0.70, 0.99, respectively. Overall competency scores between the control and intervention groups were statistically significant at P<0.001 in the pre-test with the effect size of Cohen's d= 0.68; however, in post-test I and post-test II, Cohen's d was 23.61 and 15.83, respectively.

Studies support the present findings that standardized simulation programs with high-fidelity manikins significantly improve knowledge and performance in obstetrical emergencies. One study reported an increase in knowledge scores from 36.67% to 96.67% (p<0.0004) and performance from 31.75% to 94.67% (p<0.0001), with retention scores at 91.67% after three months [16]. A similar study found that while knowledge levels were adequate (mean score 75%), skill performance was lower (54%) and declined in high-stress situations, highlighting the need for repetitive high-fidelity simulation [17]. Similarly, a clinical trial on 90 midwives showed that simulation-based education resulted in higher cognitive ability and performance in managing preeclampsia and eclampsia as compared to blended and lecture-based methods [18]. These findings emphasize that students should practice emergency management in a controlled environment and that simulation fosters confidence and competence, ultimately improving patient care outcomes.

Simulation training enhances all aspects of obstetric emergency management, including prompt diagnosis and intervention in cases such as cord prolapse, reduced diagnosis-to-delivery intervals, and improved neonatal outcomes [19]. Studies demonstrate that simulation-based learning significantly improves competency, with higher post-test knowledge (23.7 ± 4.1 vs. 22.3 ± 4.2) and skills scores (27.2 ± 4.3 vs. 24.7 ± 5.3) in the experimental group, showing statistical significance in the skill scores (t=2.65; p=0.00) [20]. A similar study reported that simulation and team-based training improved intrapartum and newborn care, with a PPH diagnosis increasing from 1.6% to 4.4% over five weeks [21]. Global organizations like WHO, FIGO, RCOG, ACOG, and Dakshata [10] have updated obstetric emergency management guidelines, emphasizing evidence-based practices. The present study reinforces that simulation training significantly improves competency in managing obstetric emergencies, supporting its integration into nursing curricula. Thus, the hypothesis H₂, stating a significant difference in competency (knowledge & skills) between control and Intervention groups, was accepted.

The present study found that 71.4% of participants were highly satisfied, while 29.5% were satisfied with obstetrical emergency simulation learning. These findings align with a cross-sectional study where students assisted by teachers during skills practice were 5.6 times more satisfied than those without assistance (AOR=5.62; 95% CI: 2.36-13.40, p<0.001) [22]. Similarly, a study on midwifery students reported high satisfaction with perinatal simulation-based training and stability over time [23]. To enhance satisfaction, improved teacher support, alignment of teaching methods with learning styles, and increased skills, practice sessions per semester are recommended to optimize simulation-based education.

Despite the positive outcomes, this study had certain limitations. The study was conducted in selected nursing colleges in Chennai, which may limit the generalizability of the findings to other regions. Additionally, the study used a moderate-fidelity simulation rather than a high-fidelity simulation, which could have further enhanced the realism of the training. Future research should consider a broader sample, multiple locations, and a longer follow-up period to evaluate knowledge and skill retention over time.

## Conclusions

The study underscores the importance of incorporating structured simulation-based training into midwifery education to enhance students' competency in obstetric emergencies. Nursing curricula should integrate standardized simulation scenarios aligned with national and international guidelines to ensure uniform training across institutions. Additionally, periodic refresher courses and competency-based assessments should be implemented to reinforce skills and knowledge. Healthcare institutions should invest in simulation centers and faculty development programs to enhance the quality of midwifery education, ultimately improving maternal and neonatal outcomes in clinical settings.

Simulation is a means of training to improve the management of obstetric emergencies. As in aviation and fire emergencies, the relatively low frequency and unpredictable life-threatening obstetric emergencies make simulation-based training the most appropriate method for training health providers to gain and maintain competence in managing obstetric emergencies. This modality has proven effective in improving providers’ knowledge, attitudes, and clinical skills. There is a plethora of evidence supporting the use of simulation-based training (SBT), specifically in the management of obstetrical emergencies. Therefore, simulation must be incorporated into the curriculum and implemented in all the subjects by trained and competent experts.

## Appendices

Appendix A

**Table 5:** Structured questionnaire to assess the knowledge on selected obstetrical emergencies among the undergraduate midwifery students' purpose The structured questionnaire is used by the investigator to assess the level of knowledge on selected obstetrical emergencies among undergraduate midwifery students. Instruction: Kindly go through the questions below and make a tick mark. The correct response carries one mark each. Please be frank in answering. It will be kept confidential, and anonymity will be maintained. Prepared by the Researcher with reference to the Dakshata Guidelines by the Ministry of Health & Family Welfare (April 2015), Indian Nursing Council Scope of Midwifery Practice (June 2021), and books.

1. This is the predominant symptom present in severe preeclampsia: Persistent systolic BP ≥ 160 mm Hg and diastolic BP > 110 mmHg; Jaundice; Abdominal pain; Chest pain
2. What are the warning signs of severe Pre-eclampsia: Headache, Epigastric pain, Abnormal edema & BP 160/110 mmHg; Vomiting, bleeding, and edema; Proteinuria, edema, and BP 140/90 mmHg; Albuminuria, weight gain, and BP 160/90mmHg
3. The drug of choice for the prevention and treatment of seizure activity caused by severe preeclampsia: Magnesium sulfate; Sodium bicarbonate; Calcium gluconate; Potassium phosphate
4. Which of the following is important for a nurse to teach about home care management in pre-eclampsia: Activity and diet restriction; Monitor the blood pressure 2-4 times a day; Urine strip test with midstream urine; All the above
5. As per the GOI, what is the dose of calcium supplementation recommended for the prevention of eclampsia: 6 months of pregnancy & during lactation; Throughout pregnancy & lactation; 3 months of pregnancy & lactation; Only during the first trimester of pregnancy
6. What are the conditions included in antepartum hemorrhage after 20 weeks of pregnancy: Abortion; Ectopic pregnancy; Abruptio placenta; Placenta previa; Vasa previa; Abortion placenta previa; Abruptio placenta and vasa previa; Placenta previa and abruptio placenta; Uterine rupture & vasa previa
7. Which of the following is described as premature separation of a normally implanted placenta during the second half of pregnancy, usually with severe hemorrhage: Ectopic pregnancy; Placenta previa; Abruptio placenta; Vasa previa
8. Which condition is associated with painless & revealed vaginal bleeding: Placenta previa; Vasa previa; Abruptio placenta; Ectopic pregnancy
9. Vasa previa is the condition where unsupported umbilical vessels are seen in: Succenturiate placenta; Bottledore placenta; Velamentous placenta; Circumvallate placenta
10. In this condition, shock is pronounced as it is out of proportion to the visible blood loss: Placenta previa; Vasa previa; Abruptio placenta; Ectopic pregnancy
11. Prevention of Abruptio placenta by early detection & effective therapy of: Preeclampsia GDM; Breech presentation; Multiple pregnancy
12. Prolonged labor is when true labor without delivery exceeds more than: 8 hours; 12 hours; 24 hours; 48 hours
13. Prolonged labor can be identified using: CTG; Vaginal examination; Partograph; Amniotomy
14. In which of the following conditions, the progressive descent of the presenting part is arrested due to mechanical factors in spite of good uterine contraction: Prolonged labor; Obstructed labor; Shoulder dystocia; Malpresentation
15. When the cervicograph crosses the alert line and falls in Zone 2, labor is considered _______ & ________is needed when it crosses the action line and falls in Zone 3: Abnormal & intervention; Normal & observation; Normal & intervention; Abnormal & observation
16. Which of the following will help to decrease the risk of infection during childbirth: Performing frequent vaginal examinations; Rupturing membranes as soon as possible in the first stage of labor; Routine catheterization of the bladder before childbirth; Reducing prolonged labor
17. Descent of the umbilical cord by the side of the presenting part following rupture of membranes is: Cord presentation; Cord prolapse; Occult prolapse; Vasa previa
18. The following are the etiology for cord prolapse except: Malpresentation; Twins; Polyhydramnios; Prolonged labor
19. The immediate complication due to the cord prolapse is: Abruptio placenta; Fetal distress; Maternal distress; IUD
20. As an initial management of cord prolapse, when immediate safe delivery is not possible: Minimize the pressure on the cord; Administer IV fluids; Refer the mother to FRU; Initiate blood transfusion
21. To prevent cord prolapse, In case of mobile head, by avoiding Vaginal examination Artificial Rupture of membranes Ambulation Administration of IV fluids
22. How to identify PPH > 500 ml of blood loss after delivery till 6 weeks after delivery: Hypotension & blood loss; Tachycardia & blood loss; Atonic uterus & blood loss
23. What are the causes for PPH (arising from pregnancy): Previous PPH; Hydramnios; Multiple pregnancy; Anemia; Abruptio placenta; Placenta previa; Iron deficiency anemia IUD; Hydrops fetalis, Polyhydramnios & previous PPH eclampsia; Previous PPH, GDM
24. The 4 “Ts” of PPH are: 1. Trauma; 2. Toxins; 3. Travel; 4. Tissue 5. Threads 6. Thrombin 7. Tears 8: Tone 1, 4, 6, & 8; 1, 2, 4, & 7; 1, 3, 5, & 6; 2, 3, 4, & 6
25. This step helps in 70% chances of preventing PPH: Observation for 2 hrs after delivery; Active management of third stage of labor; Exploration of uterovaginal canal; Examination of placenta

Appendix B 

**Table 6:** Blueprint of OSCE stations in obstetrical emergency Prepared by the Researcher with reference to the Dakshata Guidelines (April 2015), INC Scope of Midwifery Practice (June 2021) by the Ministry of Health & Family Welfare, Government of India.

	Manned	Unmanned
1	Initial management of eclampsia	Nursing responsibilities during MgSo4 administration
2	Initial management of vaginal bleeding in late pregnancy	Assessment of bleeding. Ectopic pregnancy
3	Initial management of prolonged/obstructed labor	Plotting partograph. Identifying prolonged labor
4	Initial management of cord prolapse	6. CTG tracing
5	PPH initial management of PPH: Bimanual compression, Balloon tamponade	7. Name the apparel & its purpose. 8. Drugs in PPH

Appendix C 

**Table 7:** Rating scale on satisfaction with obstetrical emergency simulation learning Developed by the researcher

S.No	Items	Highly Satisfied (4)	Satisfied (3)	Dissatisfied (2)	Highly Dissatisfied (1)
	The approach of the researcher.				
	Are you able to understand the simulation program?				
	The questions asked for assessing the knowledge of obstetrical emergencies are relevant.				
	The OSCE method of evaluation was used to assess the skills in managing obstetrical emergencies.				
	The simulation program is helpful for your future.				
	The duration of the program is adequate.				
	The simulation learning module provided enough information to provide direction and encouragement.				
	The simulation was designed for my specific level of knowledge and skills.				
	The demonstration of the simulation scenario resembled a real-life situation.				
	I had the chance to work with my peers during the simulation.				
	I felt supported by the facilitator’s assistance during the simulation.				
	The simulation allowed me to analyze my own behaviour & actions.				
	The feedback provided was timely and constructive.				
	Using simulation activities made my learning time more productive.				
	I am confident that this simulation helps in developing the skills to perform necessary tasks in a clinical setting.				
